# Supplementary material for: Targeting anti-apoptotic pathways eliminates senescent melanocytes and leads to nevi regression
Source: Nat Commun. 2022 Dec 23;13:7923. doi: 10.1038/s41467-022-35657-9 (PMC9789033; doi:10.1038/s41467-022-35657-9)
Supplement: Supplementary file 1 — Supplementary Information [file 41467_2022_35657_MOESM1_ESM.pdf]

**Targeting anti-apoptotic pathways eliminates senescent melanocytes and leads to nevi regression**

Jaskaren Kohli[1], Chen Ge[1], Eleni Fitsiou[1], Miriam Doepner[2], Simone M. Brandenburg[1], William J. Faller[3], Todd W. Ridky[2], Marco Demaria[1,*]

[1] European Research Institute for the Biology of Ageing; University Medical Center Groningen; Groningen, 9713 AV; Netherlands
[2] Department of Dermatology, Perelman School of Medicine, University of Pennsylvania, Philadelphia, PA 19104; USA
[3] Netherlands Cancer Institute; Amsterdam, 1066 CX; Netherlands

* Correspondence: m.demaria@umcg.nl

## Supplementary figures 1-4

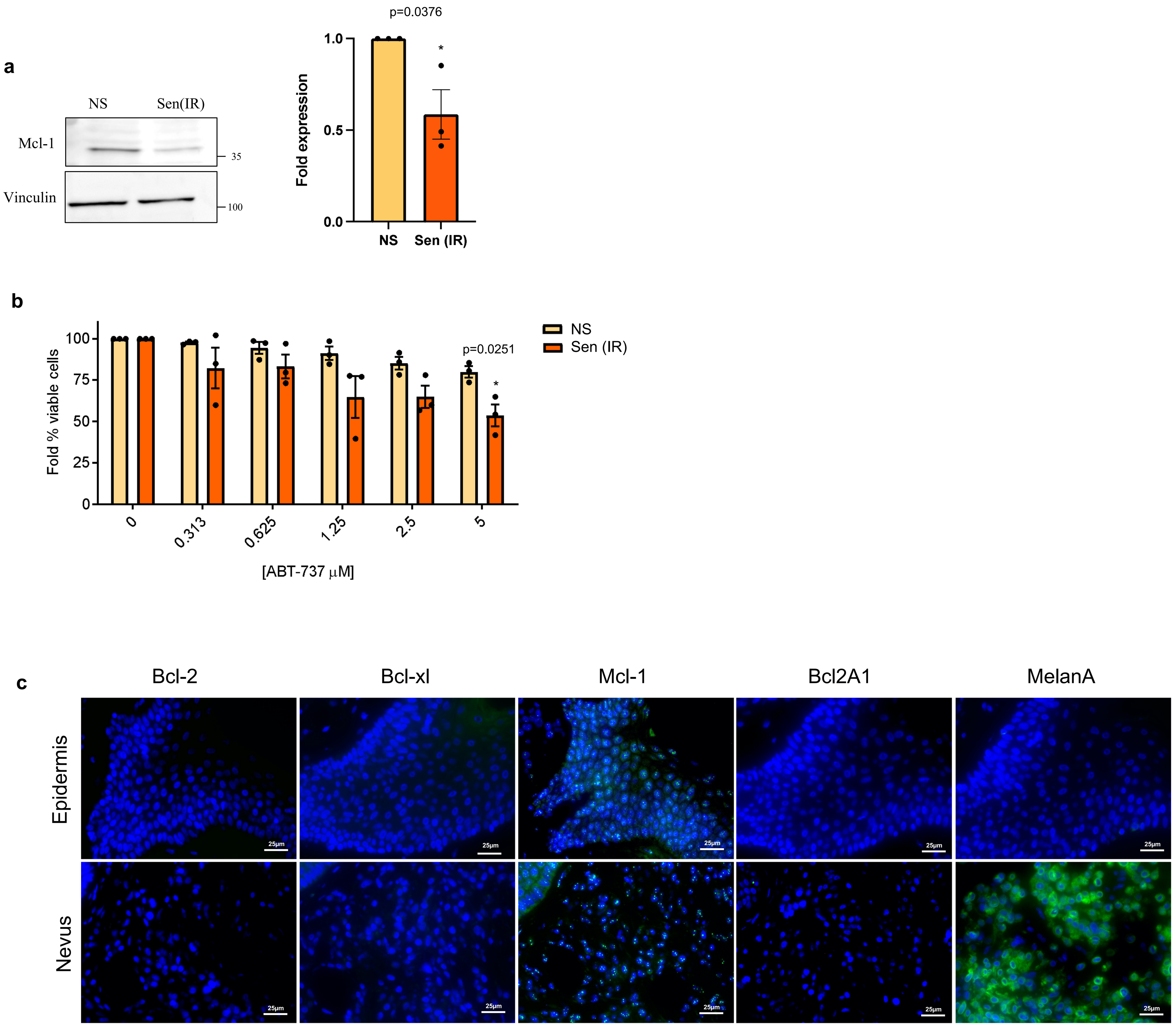


**Supplementary Figure 1 (supporting Figure 1). Senescent melanocytes are resistant to ABT-737.** (A) Protein expression and densitometry analysis of Mcl-1 from irradiated melanocytes. Vinculin was used as a housekeeping protein and expression was normalised to protein levels in non-irradiated non-senescent cells. (B) Viability of non-irradiated (NS) and irradiated (Sen IR) melanocytes treated with indicated concentrations of ABT-737 for 24 hours. Viability was normalised to untreated cells. (C) Immunofluorescence of Bcl-2, Bcl-xl, Mcl-1 and Bcl2A1 positive cells in human nevi. Melan-a was used to confirm identification of melanocytes or nevus cells, Scale bar = 25 μM. For (A), (B) and (C), n = 3 independent experiments. Error bars = S.E.M. *p<0.05. For (A) and (B), a two-tailed student's t-test was used.

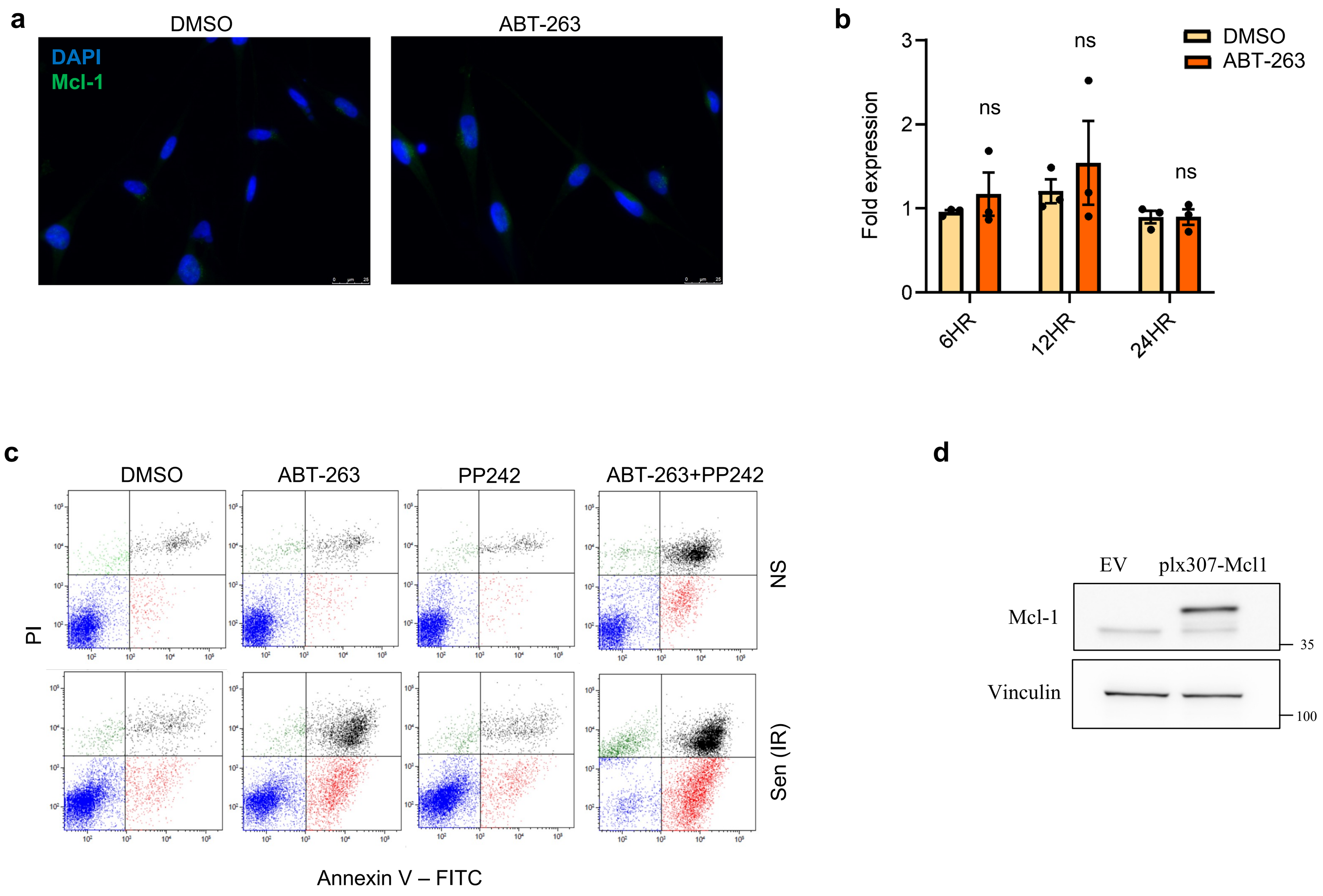


**Supplementary Figure 2 (supporting Figure 2). Pharmacological inhibition of mTOR inhibits Mcl-1 mediated resistance to ABT-263 in senescent melanocytes.** (A) Representative immunofluorescence of Mcl-1 in senescent melanocytes treated for 24 hours with DMSO or ABT-263, Scale bar = 25 μM. Quantification can be found in figure 2c. (B) mRNA analysis of *Mcl-1* from irradiated melanocytes treated with DMSO or 5μM ABT-263 for 6, 12 or 24 hours. Expression was normalised using Cq values of *tubulin*. n = 3 independent experiments. (C) Annexin V/Pi flow cytometric analysis of non-senescent (NS) and senescent (Sen IR) melanocytes treated with DMSO, 5 μM ABT-263, 1 μM PP242, or 2.5 μM ABT-263/1 μM PP242 for 24 hours. (D) Immunoblot confirming Mcl-1 overexpression in melanocytes transduced with a plx307-Mcl1 vector compared to empty vector (EV) transduced cells. Error bars = S.E.M. For (B), a two-tailed student's t-test was used.

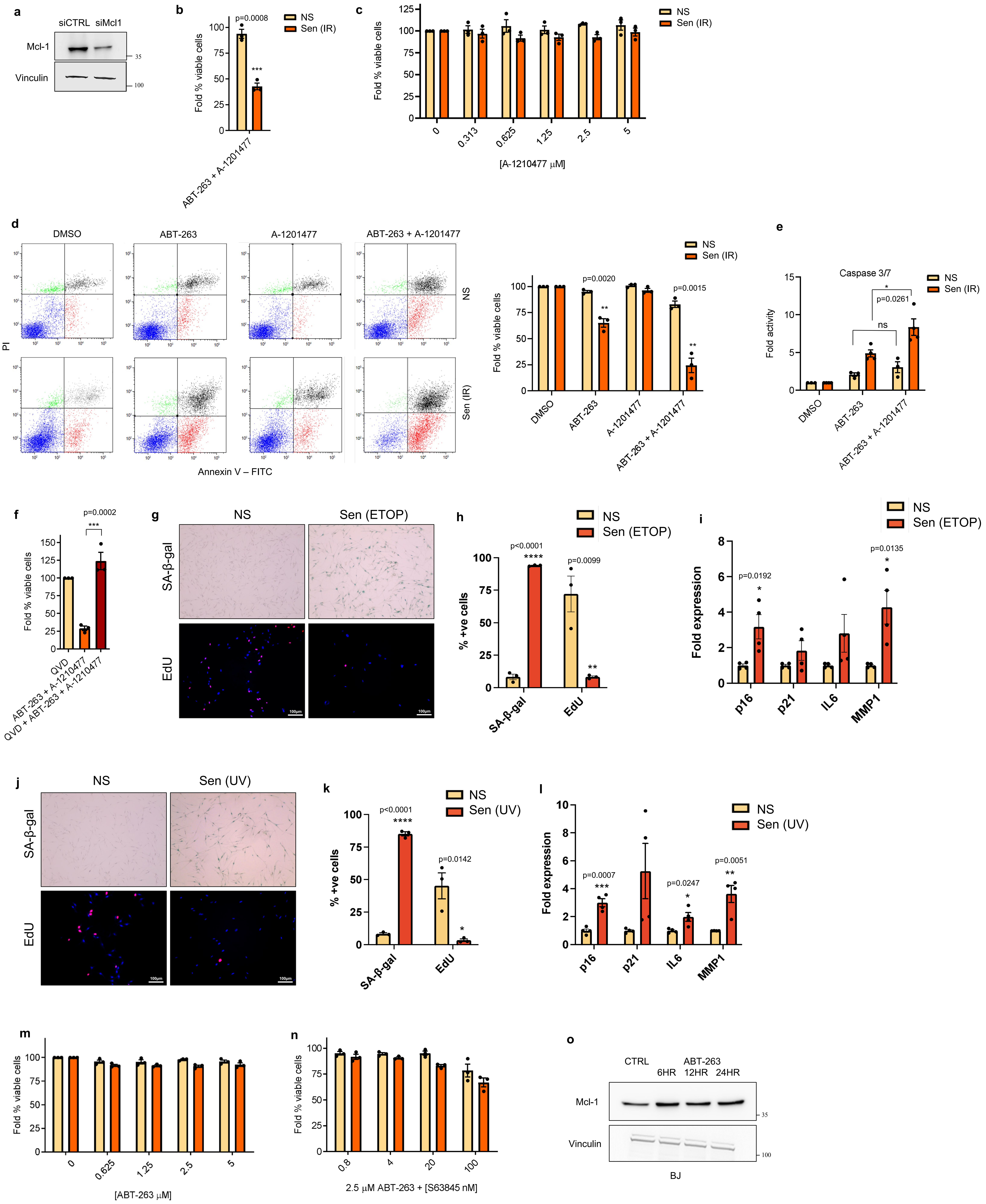


**Supplementary figure 3 (supporting Figure 3). The Mcl-1 inhibitor A-1210477 also synergises with ABT-263 to induce apoptosis in senescent melanocytes.** (A) Immunoblot demonstrating confirmation of reduced Mcl-1 expression in siMCl-1 transfected cells. Viability of non-irradiated (NS) and irradiated (Sen IR) melanocytes treated with (B) 2.5 µM ABT-263/ 2.5 µM A-1210477 or (C) only A-1210477 for 24 hours. (D) Annexin V/Pi flow cytometric analysis and quantification of non-irradiated non-senescent and irradiated senescent melanocytes treated with DMSO, 5 µM ABT-263, 5µM A-1201477 or 2.5 µM ABT-263/2.5 µM A-1210477 for 24 hours. The percentage of viable cells in drug treated cells were normalised to the percentage of DMSO treated viable cells. (E) Caspase 3/7 activity in non-irradiated and irradiated melanocytes treated with DMSO, 5 µM ABT-263, or 2.5 µM ABT-263/2.5 µM A-1210477 for 24 hours. (F) Viability of irradiated melanocytes treated with 20 µM QVD, 2.5µM ABT-263/2.5 µM A-1210477, or 20 µM QVD/2.5µM ABT-263/2.5 µM A-1210477 for 24 hours. Viability was normalised to untreated cells. (G) SA-β-gal/EdU images and (H) quantification of DMSO treated (NS) and etoposide treated (Sen ETOP) melanocytes, Scale bar = 100 µM. (I) mRNA analysis of *p16*, *p21*, *IL6* and *MMP1*. (J) SA-β-gal/EdU images and (K) quantification of non-irradiated (NS) and UV-irradiated treated (Sen UV) melanocytes, Scale bar = 100 µM. (L) mRNA analysis of *p16*, *p21*, *IL6* and *MMP1*. Viability of non-irradiated (NS) and irradiated (Sen IR) BJ fibroblasts treated with indicated concentrations of (M) ABT-263 and/or (N) ABT-263/S63845. (O) Protein expression of Mcl-1 in irradiated BJ cells treated with 5 µM ABT-263 for indicated time points. qPCR expression was normalised using Cq values of tubulin in all experiments. Error bars = S.E.M. *p<0.05, **p<0.01, ***p<0.001. For all panels significance was carried out with a two-tailed student's t-test. For all panels, n = 3 independent experiments except 3i and 3l where n = 4 independent experiments.

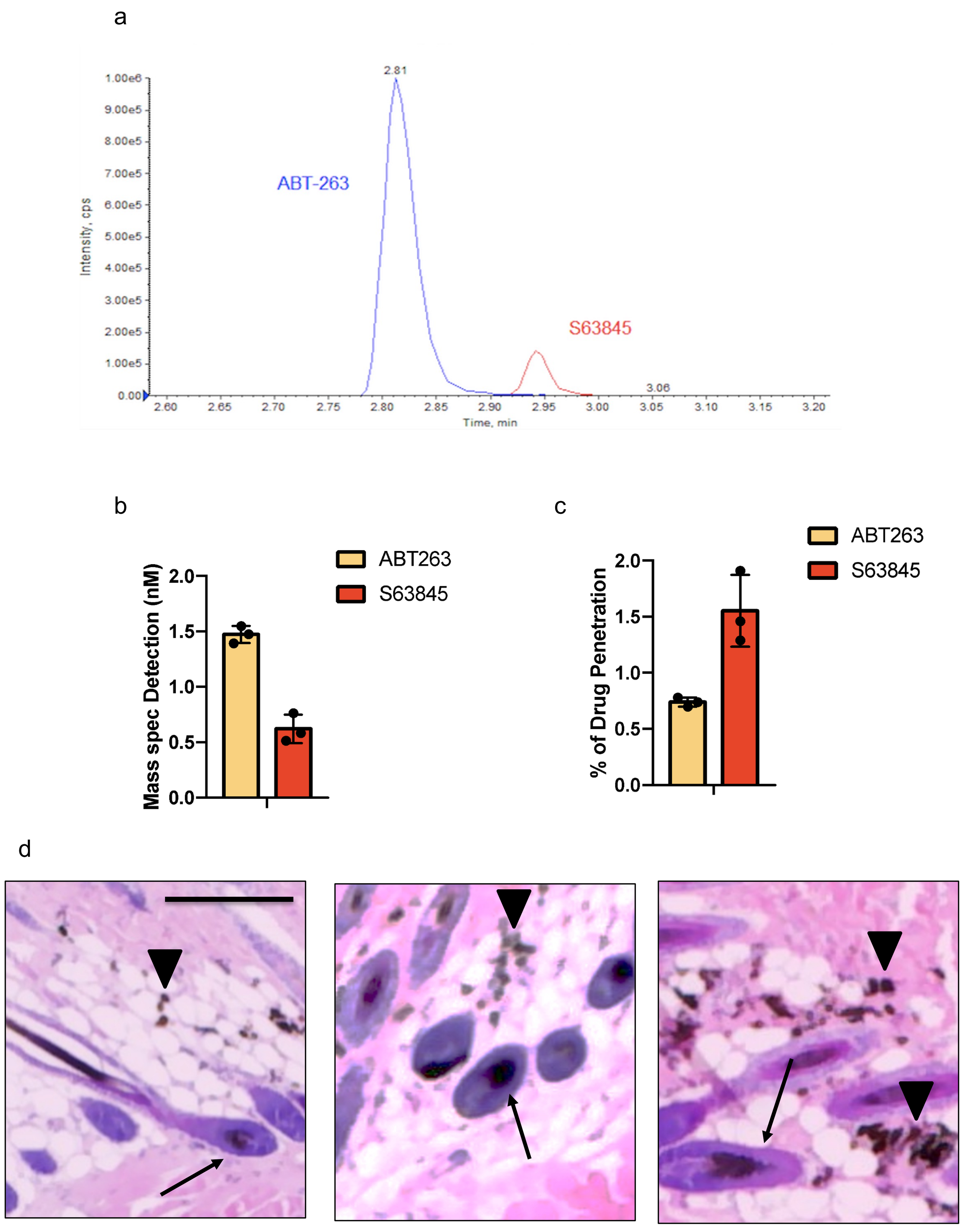


**Supplementary figure 4. Topical application of ABT-263 and S63845 does not kill follicular melanocytes. (a)** A mass chromatogram of ABT-263 and S63845 of mouse skin biopsy. (b) Molar mass of drugs ABT-263 and S63845 detected from skin biopsy, N=3. (c) The percentage of the drug penetration the skin, calculated as Molar mass of drug detected from skin biopsy divided by total molar mass of topical administration, N=3. Error bars = S.D. (D) H and E images of a hair follicle containing follicular pigmented melanocytes (arrow) in mice topically treated with ABT-263 and S63845. Arrowheads show nevi remnants post treatment. The images are representative of 10 individual mice. Scale bar = 250μM.

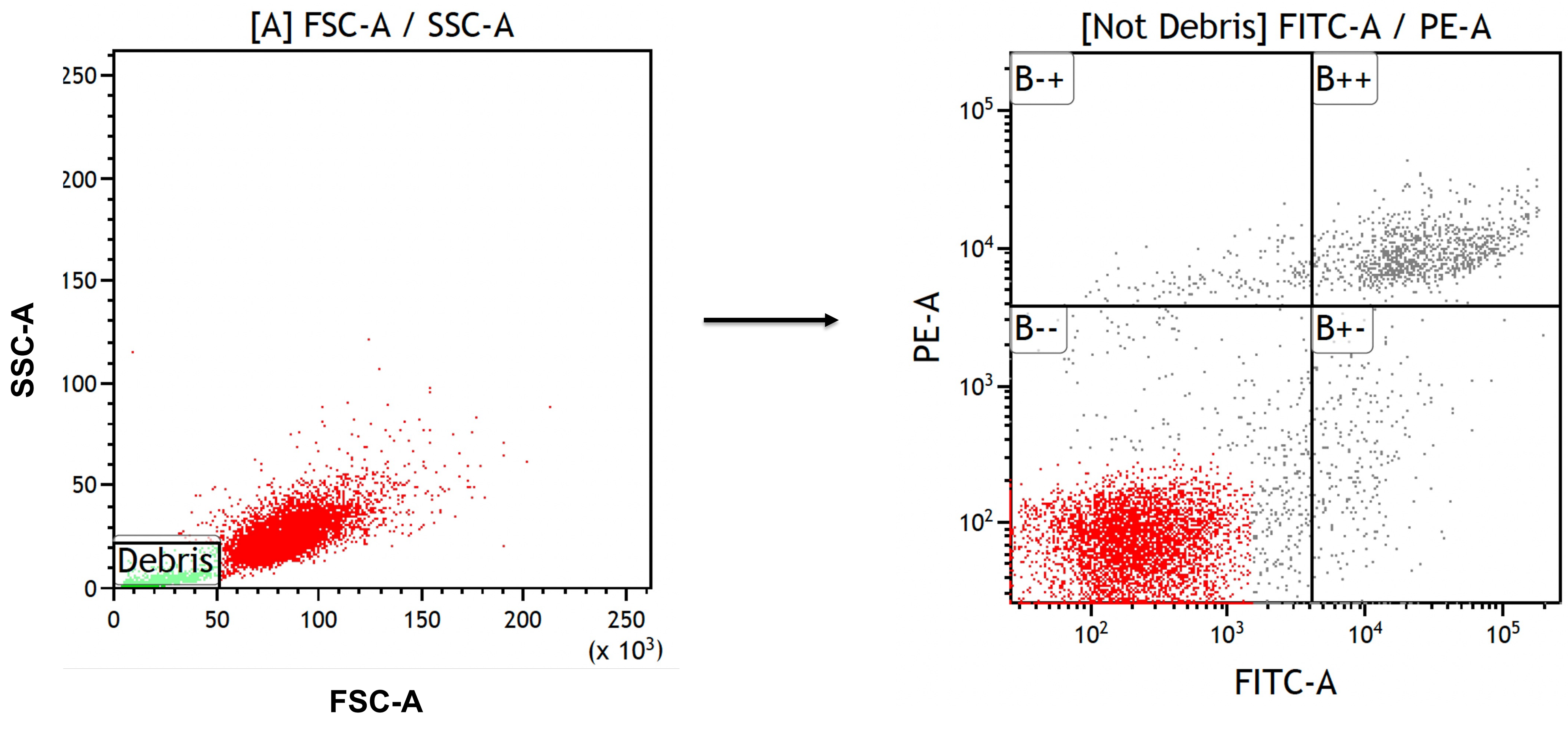


1) Gate out debris and only gate on live cells (red)

2) Analyse Annexin-FITC and PI positivity on live (non debris) cells.

**Supplementary figure 5. Gating strategy for Annexin-Pi flow cytometry experiments.** Debris was first gated out by gating out events with low FSC-A and low SSC-A. Gated cells (red) were then analysed for Annexin V-FITC and PI. Live cells were gated as low annexin V-FITC and low PI (B--). Early apoptotic cells were gated as high Annexin V-FITC and low PI (B+-) and late apoptotic cells were gated as high annexin V-FITC and high PI (B++).

**Uncropped blots in supplementary figures.**

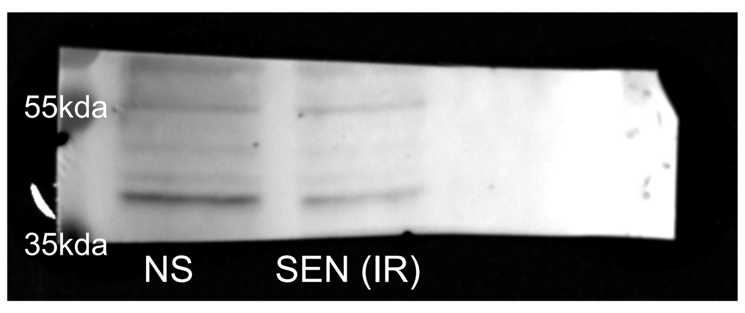

Mcl-1 Supp Figure 1a

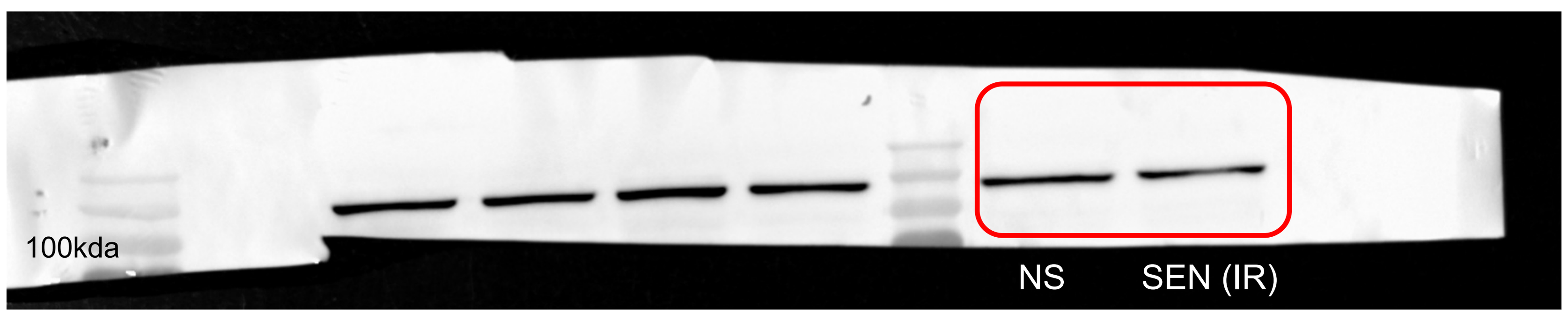

Vinculin Supp Figure 1a

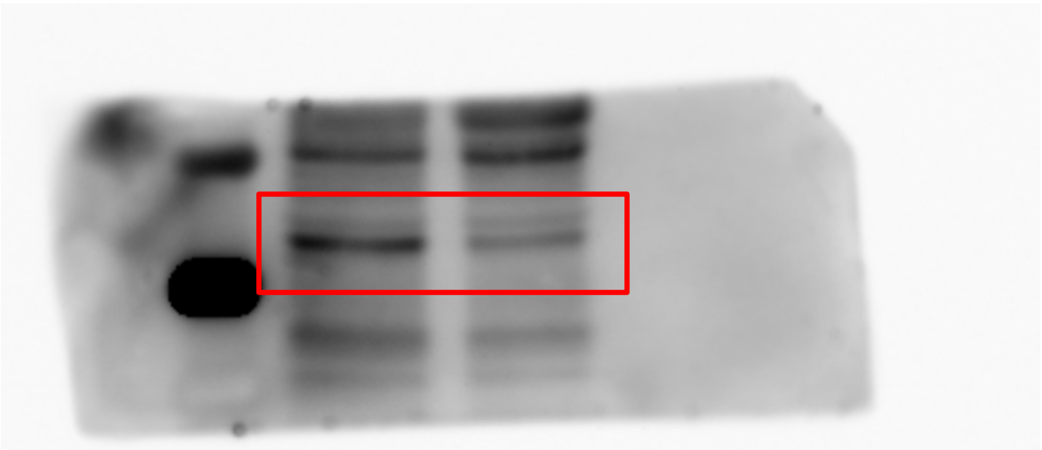
Mcl-1 Supp Figure 1a (repeat 2)

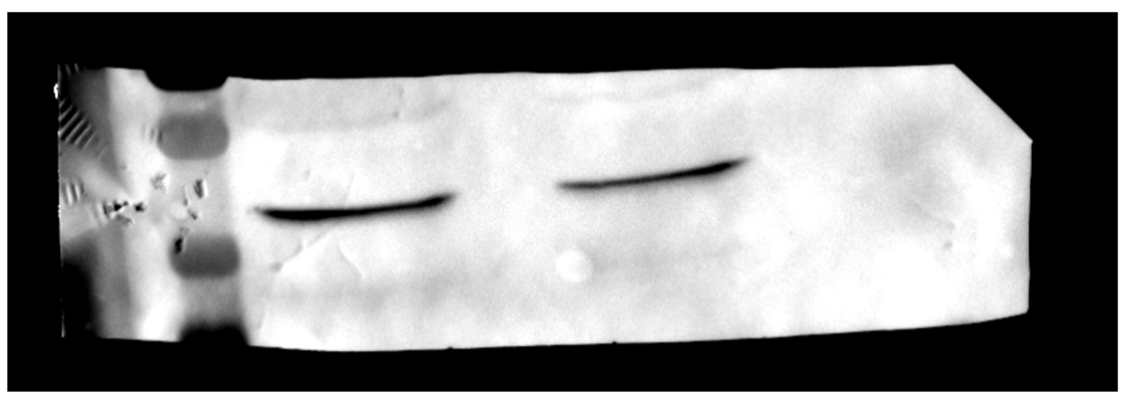
Mcl-1 Supp Figure 1a (repeat 3)

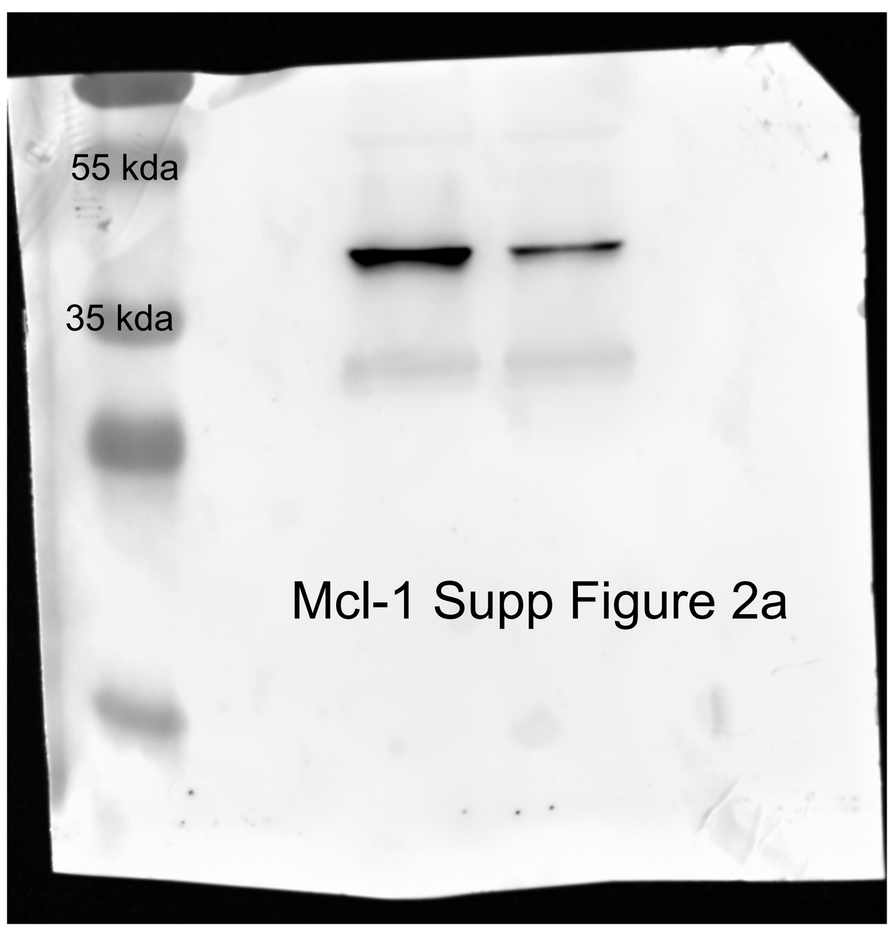

Mcl-1 Supp Figure 2a

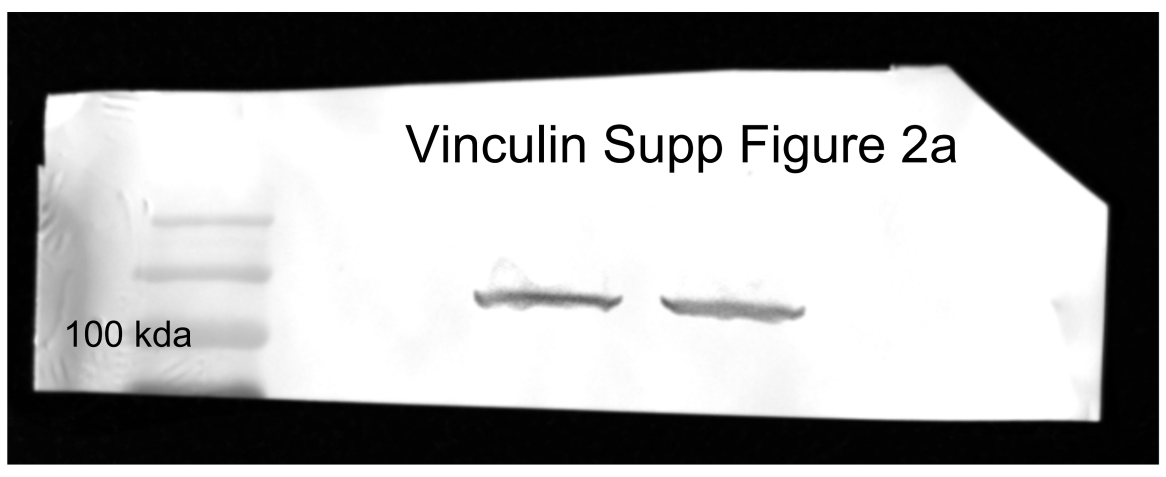

Vinculin Supp Figure 2a

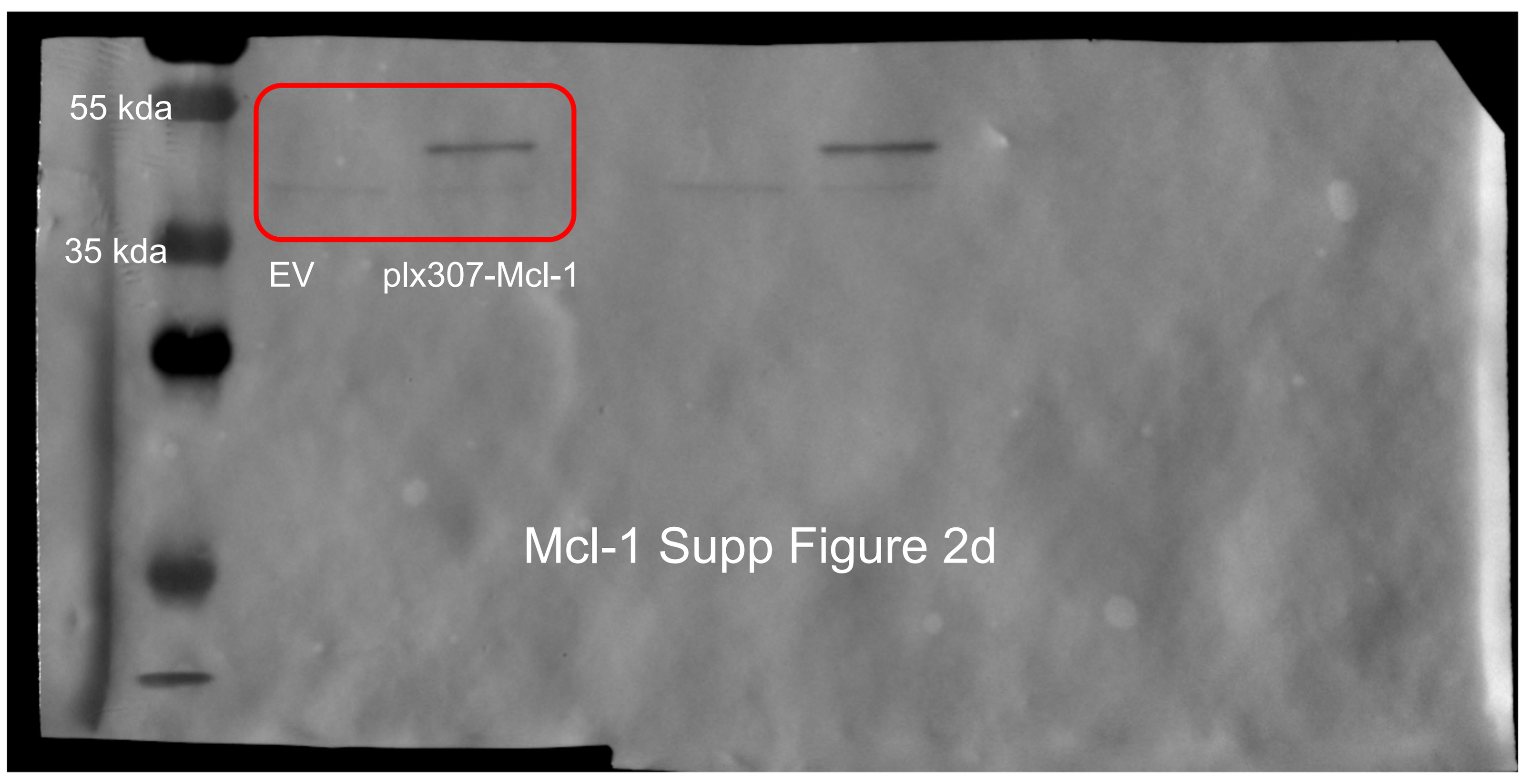

Mcl-1 Supp Figure 2d

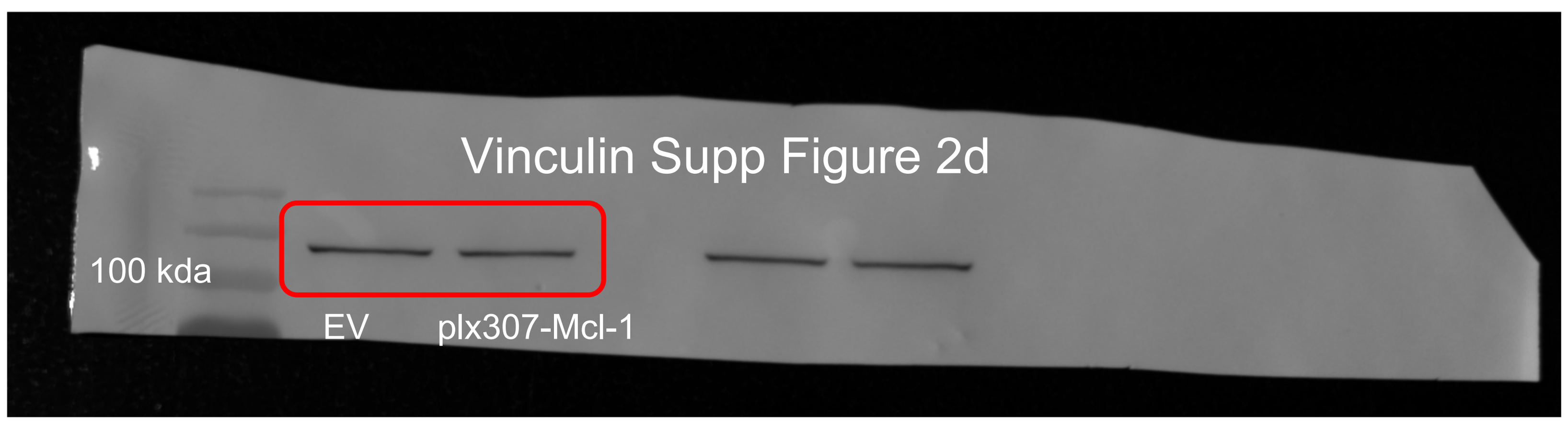

Vinculin Supp Figure 2d

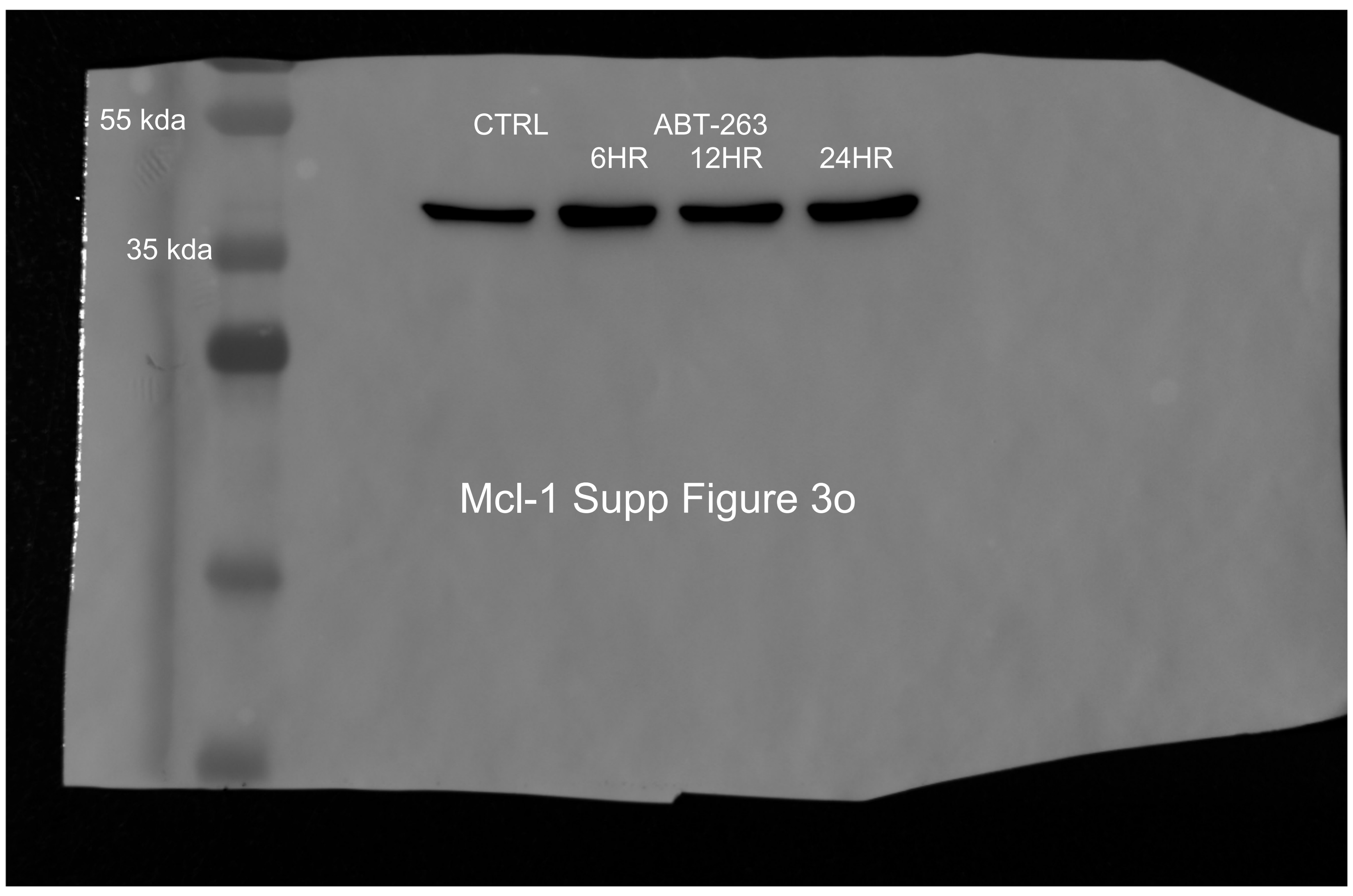

Mcl-1 Supp Figure 3o

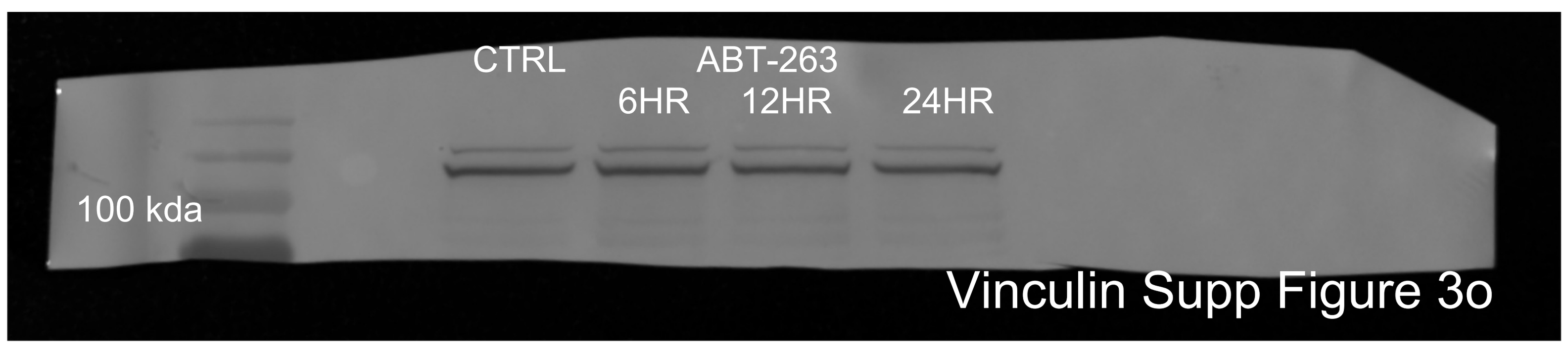

Vinculin Supp Figure 3o
