# Supplementary material for: Targeting anti-apoptotic pathways eliminates senescent melanocytes and leads to nevi regression
Source: Nat Commun. 2022 Dec 23;13:7923. doi: 10.1038/s41467-022-35657-9 (PMC9789033; doi:10.1038/s41467-022-35657-9)
Supplement: Supplementary file 3 — Reporting Summary [file 41467_2022_35657_MOESM3_ESM.pdf]

nature portfolio

Corresponding author(s): Marco Demaria

Last updated by author(s): Dec 6, 2022

Reporting Summary

Nature Portfolio wishes to improve the reproducibility of the work that we publish. This form provides structure for consistency and transparency in reporting. For further information on Nature Portfolio policies, see our Editorial Policies and the Editorial Policy Checklist.

Statistics

For all statistical analyses, confirm that the following items are present in the figure legend, table legend, main text, or Methods section.

n/a	Confirmed	
☐	☒	The exact sample size (*n*) for each experimental group/condition, given as a discrete number and unit of measurement
☐	☒	A statement on whether measurements were taken from distinct samples or whether the same sample was measured repeatedly
☐	☒	The statistical test(s) used AND whether they are one- or two-sided<br>*Only common tests should be described solely by name; describe more complex techniques in the Methods section.*
☐	☒	A description of all covariates tested
☐	☒	A description of any assumptions or corrections, such as tests of normality and adjustment for multiple comparisons
☐	☒	A full description of the statistical parameters including central tendency (e.g. means) or other basic estimates (e.g. regression coefficient) AND variation (e.g. standard deviation) or associated estimates of uncertainty (e.g. confidence intervals)
☐	☒	For null hypothesis testing, the test statistic (e.g. *F*, *t*, *r*) with confidence intervals, effect sizes, degrees of freedom and *P* value noted<br>*Give P values as exact values whenever suitable.*
☒	☐	For Bayesian analysis, information on the choice of priors and Markov chain Monte Carlo settings
☒	☐	For hierarchical and complex designs, identification of the appropriate level for tests and full reporting of outcomes
☒	☐	Estimates of effect sizes (e.g. Cohen's *d*, Pearson's *r*), indicating how they were calculated

Our web collection on statistics for biologists contains articles on many of the points above.

Software and code

Policy information about availability of computer code

Data collection	No software was used.
Data analysis	Statistical analysis was carried out with Graphpad Prism version 8. Flow cytometry data was analysed using Kaluza flow cytometry analysis software version 2.0. (Beckman). Immunoblots were analysed with ImageQuant Software TL (GE Healthcare). Nevus density and immunoflourescence images were analysed using ImageJ version 1.53.

For manuscripts utilizing custom algorithms or software that are central to the research but not yet described in published literature, software must be made available to editors and reviewers. We strongly encourage code deposition in a community repository (e.g. GitHub). See the Nature Portfolio guidelines for submitting code & software for further information.

Data

Policy information about availability of data

All manuscripts must include a data availability statement. This statement should provide the following information, where applicable:

- Accession codes, unique identifiers, or web links for publicly available datasets
- A description of any restrictions on data availability
- For clinical datasets or third party data, please ensure that the statement adheres to our policy

Authors can confirm that all relevant data are included in this article and/or its supplementary information files. Source data are provided as a source data file. Data are available from the corresponding author (m.demaria@umcg.nl) upon reasonable request

Human research participants

Policy information about studies involving human research participants and Sex and Gender in Research.

Reporting on sex and gender	N/A
Population characteristics	N/A
Recruitment	N/A
Ethics oversight	N/A

Note that full information on the approval of the study protocol must also be provided in the manuscript.

Field-specific reporting

Please select the one below that is the best fit for your research. If you are not sure, read the appropriate sections before making your selection.

- [x] Life sciences
- [ ] Behavioural & social sciences
- [ ] Ecological, evolutionary & environmental sciences

For a reference copy of the document with all sections, see nature.com/documents/nr-reporting-summary-flat.pdf

Life sciences study design

All studies must disclose on these points even when the disclosure is negative.

Sample size	Sample sizes are based on previous experiments where typically three to four independent samples are required for statistical purposes.
Data exclusions	No data were excluded.
Replication	All experiments were reproduced and independent numbers of experiments are written in each figure legend.
Randomization	Animals were randomly assigned for each treatment group.
Blinding	Quantifications from immunohistochemistry and immunoflourescence experiments were blinded and analyzed by an independent second investigator.

Reporting for specific materials, systems and methods

We require information from authors about some types of materials, experimental systems and methods used in many studies. Here, indicate whether each material, system or method listed is relevant to your study. If you are not sure if a list item applies to your research, read the appropriate section before selecting a response.

Materials & experimental systems

n/a	Involved in the study	
[ ]	[x]	Antibodies
[ ]	[x]	Eukaryotic cell lines
[x]	[ ]	Palaeontology and archaeology
[ ]	[x]	Animals and other organisms
[x]	[ ]	Clinical data
[x]	[ ]	Dual use research of concern

Methods

n/a	Involved in the study	
[x]	[ ]	ChIP-seq
[ ]	[x]	Flow cytometry
[x]	[ ]	MRI-based neuroimaging

Antibodies

Antibodies used

Bcl-2, Cell Signalling Technology, Cat#15071, Clone#124
Bcl-xL, Cell Signalling Technology, Cat#2764, Clone#54H6, Lot#9
Bcl-w, Cell Signalling Technology, Cat#2724, Clone#31H4, Lot#5
Mcl-1, Cell Signalling Technology, Cat#94296, Clone#D2W9E, Lot#1
Mcl-1, Cell Signalling Technology, Cat#sc-12756, Clone#22, Lot#D0318
p-p70s6K, Cell Signalling Technology, Cat#9234, Clone#108D2, Lot#12
p70S6K, Cell Signalling Technology, Cat#9202, Lot#20

	Vinculin, Sigma-Aldrich, Cat#V9131, Clone#hVIN-1<br>Bcl-w, Proteintech, Cat#16026-1-AP<br>MelanA, Abcam, Cat#ab210546, Clone#EPR20380, Lot#GR303449-4<br>MelanA/MART(NCL-L-MelanA), Leica Biosystems, Cat#PA0233, Clone#A103<br>β-Actin, Cell Signalling Technology, Cat#3700, Clone#8H10D10<br>BRAFV600E, Signal-Aldrich, Cat#SAB5600047, Clone#RM8
Validation	Antibody validation can be found on the manufacturer's website.

Eukaryotic cell lines

Policy information about cell lines and Sex and Gender in Research

Cell line source(s)	Melanocytes and IMR-90 fibroblasts were both obtained from ATCC.
Authentication	Cells were authenticated by the ATCC.
Mycoplasma contamination	Cell lines were regularly tested negative for mycoplasma.
Commonly misidentified lines (See ICLAC register)	No commonly misidentified cell lines were used.

Animals and other research organisms

Policy information about studies involving animals; ARRIVE guidelines recommended for reporting animal research, and Sex and Gender in Research

Laboratory animals	B6.Cg-Tg(Tyr-cre/ERT2)13Bos/J animals were obtained from the Jackson laboratory. BRAFV600E mice were obtained from Christian Blank (Netherlands Cancer Institute). Animals were bred to obtain required genotypes. Male, nine week old mice were used.
Wild animals	No wild animals were used.
Reporting on sex	Only males were used as females do not induce hyperpigmented lesions at similar rates.
Field-collected samples	Field-collected animals were not used.
Ethics oversight	This study was approved by the Animal Welfare body in the University Medical Centre Groningen.

Note that full information on the approval of the study protocol must also be provided in the manuscript.

Flow Cytometry

Plots

Confirm that:

- [x] The axis labels state the marker and fluorochrome used (e.g. CD4-FITC).
- [x] The axis scales are clearly visible. Include numbers along axes only for bottom left plot of group (a 'group' is an analysis of identical markers).
- [x] All plots are contour plots with outliers or pseudocolor plots.
- [x] A numerical value for number of cells or percentage (with statistics) is provided.

Methodology

Sample preparation	Treated melanocytes were processed as described in the Dead Cell Apoptosis Kit with Annexin V (ThermoFisher Scientific).
Instrument	BD FACSCanto II
Software	Kaluza Flow cytometry analysis software.
Cell population abundance	Early apoptotic cells were defined by cells displaying high annexin V and low PI Flourecence. Late apoptotic cells were defined as cells displaying high annexin V and high PI flourescence. Viable cells were defined as cells displaying low Annexin V and low PI levels.
Gating strategy	FSC/SSC gates were first made to exclude cellular debris (low FSC/SSC). Gated cells were then analyzed for Annexin V and PI levels.

- [x] Tick this box to confirm that a figure exemplifying the gating strategy is provided in the Supplementary Information.
